# Anti-Leukemia Activity of *In Vitro*-Expanded Human Gamma Delta T Cells in a Xenogeneic Ph^+^ Leukemia Model

**DOI:** 10.1371/journal.pone.0016700

**Published:** 2011-02-03

**Authors:** Gabrielle M. Siegers, Tania C. Felizardo, A. Mark Mathieson, Yoko Kosaka, Xing-Hua Wang, Jeffrey A. Medin, Armand Keating

**Affiliations:** 1 Cell Therapy Program, Princess Margaret Hospital, University Health Network, Toronto, Ontario, Canada; 2 Ontario Cancer Institute, Toronto, Ontario, Canada; Emory University, United States of America

## Abstract

Gamma delta T cells (GDTc) lyse a variety of hematological and solid tumour cells *in vitro* and *in vivo*, and are thus promising candidates for cellular immunotherapy. We have developed a protocol to expand human GDTc *in vitro*, yielding highly cytotoxic Vgamma9/Vdelta2 CD27/CD45RA double negative effector memory cells. These cells express CD16, CD45RO, CD56, CD95 and NKG2D. Flow cytometric, clonogenic, and chromium release assays confirmed their specific cytotoxicity against Ph^+^ cell lines *in vitro*. We have generated a fluorescent and bioluminescent Ph^+^ cell line, EM-2eGFPluc, and established a novel xenogeneic leukemia model. Intravenous injection of EM-2eGFPluc into NOD.Cg-*Prkdcscid Il2rgtm1Wjl*/SzJ (NSG) mice resulted in significant dose-dependent bone marrow engraftment; lower levels engrafted in blood, lung, liver and spleen. *In vitro*-expanded human GDTc injected intraperitoneally were found at higher levels in blood and organs compared to those injected intravenously; GDTc survived at least 33 days post-injection. In therapy experiments, we documented decreased bone marrow leukemia burden in mice treated with GDTc. Live GDTc were found in spleen and bone marrow at endpoint, suggesting the potential usefulness of this therapy.

## Introduction

Gamma delta T cells (GDTc) are immunosurveillance cells, involved in both innate and adaptive immunity. GDTc are promising candidates for adoptive immunotherapy because they elicit cytolytic responses against a variety of allogeneic and autologous tumors *in vitro* and *in vivo* [1], [2], [3], [4], [5]. Most GDTc circulating in the peripheral blood are of the Vγ9Vδ2 subset; they recognize and are activated by a number of hematological malignancies [6], [7], [8], [9]. Indeed, disease status in acute myeloid leukemia patients was found to correlate with circulating GDTc levels [10]. Likewise, elevated GDTc counts correlated with improved disease-free survival in leukemia patients following T cell-depleted allogeneic bone marrow transplantation [11]; furthermore, this GDTc increase was sustained over several years [12].

A pilot study adoptively transferring autologous GDTc into patients with advanced renal cell carcinoma by Kobayashi et al was without severe adverse effects and showed that an increase in peripheral blood GDTc correlated with prolonged tumor doubling times [13]. The same group recently reported on a patient in complete remission two years after autologous, *in vitro*-expanded GDTc therapy, which eradicated multiple lung metastases [14]. In a recent Phase I trial, autologous *ex vivo*-expanded GDTc were infused into patients with recurrent non-small-cell lung cancer; GDTc were well tolerated, however, efficacy could not be determined due to the small cohort size (n = 10) [15]. In the few patients treated to date however, infusion of autologous *in vitro* expanded cytotoxic GDTc appears safe and may constitute novel therapy to eradicate various hematological malignancies.

Ph+ leukemia arises from the fusion of *bcr* and *abl* genes [16], [17], [18]. Treatment with imatinib mesylate (IM), a tyrosine kinase inhibitor (TKI) that targets p210^Bcr-Abl^ [19] constitutes standard of care for newly diagnosed CML patients [20]. While the majority achieves a complete cytogenetic response and the restoration of normal hematopoiesis, lifetime therapy with TKIs is required for most because quiescent malignant CML clones are not eradicated by the treatment [21]. Although second- and third-generation TKIs may offer improved efficacy over IM, none are yet able to definitively cure CML [22]. Mustjoki recently reported eradication of most Ph+ progenitors in chronic phase CML patients on TKIs, but suggested that anti-CML immune control dictates remission in patients discontinuing TKI therapy [23]. Thus, it is important to develop novel therapeutic approaches that bolster the immune system to attain complete disease eradication. This approach is especially important for patients presenting with TKI-refractory disease. CML is responsive to immunotherapeutic approaches, as evidenced by positive outcomes after donor lymphocyte infusion and earlier studies with interferon alpha. Furthermore, vaccination of CML patients with a multipeptide targeting the p210 fusion protein improved cytogenetic responses [24]. Moreover, immunotherapy that can eliminate TKI-induced minimal disease offers the possibility of discontinuing therapy and perhaps cure.

Kreutzman et al recently showed that clonal lymphocytes, including GDTc, existing in CML patients at diagnosis specifically expand in the context of dasatinib therapy [25]. In conjunction with earlier studies correlating clonal lymphocyte expansions with positive clinical outcome [26], this suggests an anti-leukemia role for clonal GDTc *in vivo*. We have developed a protocol to expand human GDTc *in vitro* and confirmed their selective cytotoxicity to Ph^+^ leukemia cell lines. We have also established a novel xenogeneic model with bioluminescent leukemia cells to test GDTc therapy *in vivo.*

## Materials and Methods

### Ethics statement

Human blood samples were collected from healthy adult donors after obtaining written informed consent according to UHN Research Ethics Board approved protocols. This study was approved by the institutional Animal Care Committee of the University Health Network (Permit Number: 917.9), and carried out in accordance with the Canadian Council on Animal Care Guidelines.

### Cells

#### Primary GDTc

Peripheral blood mononuclear cells (PBMCs) were recovered using density gradient separation (Ficoll-Paque, GE Healthcare, Piscataway NJ). GDTc were enriched from the PBMC fraction by magnetic sorting using the Miltenyi γδ T cell positive selection kit (Miltenyi Biotec, Auburn CA) as per the manufacturer's instructions. GDTc were eluted into complete medium containing 1 µg/ml Concanavalin A (ConA, Sigma-Aldrich, Oakville ON) then plated at 2.5×10^5^/ml in 24-well plates. Complete medium: AIM-V (Gibco) with 5% human AB serum (Lonza, Walkersville, MD), 10 ng/ml each recombinant human IL-2 (rIL-2, Proleukin, Novartis Pharmaceuticals, Canada) and IL-4 (R&D Systems, Minneapolis, MN). Donor cells were cultured for 6–8 days in ConA, reaching exponential growth. They were then harvested, media containing ConA was removed and GDTc were further cultured in complete medium (above) without ConA. Cell enumeration and viability were assessed by use of a hemocytometer and Trypan blue exclusion. Cells were re-plated in fresh complete medium at 2.5×10^5^/ml or 5.0×10^5^/ml in 24-well plates and maintained in a humidified atmosphere at 37°C with 5% CO_2_.

#### Cell Lines

The Ph^+^ myeloid cell line K562 and Burkitt Lymphoma line Raji were obtained from the American Type Culture Centre (ATCC) (Manassas, VA). The Ph+ myeloid leukemia cell line, EM-2, was derived from a patient with CML relapsing with a Ph+ myeloid blast crisis after allogeneic bone marrow transplantation; this line has been available in our lab since the 1980s [27]. EM-2eGFPluc and RAJIeGFPluc were generated as described below.

### Lentivirus Production and Cell Transduction

A lentiviral vector (LV) encoding the cDNAs for enhanced GFP and luciferase (eGFPluc) (pCCL.sin.cPPT.polyA.CTE.eGFP.minhCMV.hPGK.luc.WPRE) was constructed using a dual promoter system. Expression of eGFP was driven by the minimal human CMV (minhCMV) promoter and luciferase expression was driven by the human phosphoglycerate kinase (hPGK) promoter. The parent vector containing a different dual promoter system was originally obtained from Dr. L. Naldini [28]; the truncated version of low-affinity NGF receptor (ΔLNGFR) in that parent construct was replaced by the firefly luciferase cDNA by standard molecular biology techniques. Recombinant leiviral virions were generated following our protocol [29] with few modifications. Briefly, 293T cells were transiently transfected using a three-plasmid packaging system (LV plasmid construct, the packaging plasmid pCMVR8.91, and the vesicular stomatitis virus glycoprotein envelope-coding plasmid pMD.G) in the presence of polyethylenimine (PEI). Viral supernatants were harvested at 24 and 48 hours post-transfection and concentrated by ultracentrifugation. The concentrated viral supernatants were serially diluted and functionally titered on 293T cells; productively transduced cells were enumerated by flow cytometry (data not shown). A functional titer of ∼3×10^8^ infectious particles/ml was obtained after concentration. EM-2 and RAJI cells were transduced with the eGFPluc LV at an effective MOI of 1; positive cells were sorted by flow cytometry for eGFP expression, expanded and then frozen and stored in liquid nitrogen until required.

#### CD107 Assays

1×10^5^ GDTc were mixed with 5×10^5^ target cells in AIM V complete media in a 96-well round bottom plate. 5 µl anti-CD107Alexa647 (Biolegend, H4A3) was added to all wells to a final concentration of 10 µg/ml. As a positive control, 0.15 nM PMA (Sigma-Aldrich, Oakville ON) and 0.3 µg/ml Ionomycin (Sigma-Aldrich, Oakville ON) were added to one well. Cells were incubated for 1 h at 37°C. Subsequently, 8.5 µl of a 1∶50 dilution of Golgi stop (BD Biosciences) was added to a final concentration of 2.6 µM. Incubation continued at 37°C for 2 h, after which cells were placed in the dark on ice. Further staining was performed as described in ‘Flow cytometry’.

### Bioluminescent imaging (BLI)

#### Cells

EM-2eGFPluc were re-suspended in PBS such that the total cell number was 1.25×10^5^ cells in the first well, then serial dilutions were performed 1∶2 in PBS for 7 wells of a 96-well flat-bottomed plate. 100 µl luciferin was added to 100 µl cell suspension in a 96-well flat bottom to a final concentration of 150 µg/ml and imaging performed a minimum of 5 minutes later using the IVIS® imaging system (Xenogen, Alameda, CA). Regions of interest were identified and luminescence was quantified using IVIS® technology and LivingImage™ software (Xenogen). Linear regression analyses were calculated using Excel.

#### Mice

Luciferin was injected IP at 150 mg/kg 10–15 min prior to scanning. Mice were anesthetized using 3–4% isofluorane administered via the Xenogen vaporiser, until loss of consciousness and then they were maintained at 1.5–2% isofluorane. The IVIS® imaging system and Living Image™ software were used to acquire the images and quantify bioluminescence as above.

#### Flow cytometry

Fluorescence-activated cell sorting (FACS) was performed on a FACS Calibur (Becton Dickenson, Mississauga ON), calibrated with CaliBRITE Beads (Becton Dickenson, Mississauga ON), and data was analyzed with CellQuest™ Software (Becton Dickenson, Mississauga ON) where indicated. Viability was determined with 7-AAD (Sigma-Aldrich, Oakville ON, 5 µl/sample). All other flow cytometry was done using the FC-500 (Beckman-Coulter) and analysis was performed using FlowJo^©^ software.

#### Antibodies

The following antibodies were used for GDTc immunophenotyping: anti-CD3 APC (BioLegend, UCHT1, 0.5 µg/ml), anti-CD16 PE (BioLegend, 3G8, 2.5 µg/ml), anti-CD27 APC (BD Biosciences, M-T271, 0.5 µg/ml), anti-CD45RA FITC (BD PharMingen, H1 100, 5 µg/ml), anti-CD45RO PE (BD PharMingen, UCHL1, 1.25 µg/ml), anti-CD56 FITC (BioLegend, MEM-188, 5 µg/ml), anti-CD95PE (BD Biosciences, DX2, 2.5 µg/ml), anti-NKG2D PE (R&D Systems, 149810, 0.5 µg/ml), anti-TCR alpha/beta FITC (BD Biosciences, T10B9.1A-31, 0.2 µg/ml), anti-TCR alpha/beta PE (Biolegend, IP26, 2.5 µg/ml), anti-TCR gamma/delta PE (eBiosciences, B1.1, 20 µg/ml), anti-Vgamma9 biotin (1∶10, kind gift of Dr. Li Zhang, UHN) followed by streptavidin-APC (eBiosciences, 0.2 µg/ml) and anti-Vdelta2PE (BioLegend, B6, 0.25 µg/ml). Annexin V-FITC (Biovision, Mountain View CA, 5 µl = 0.75 µg/sample) and 7-AAD (Sigma-Aldrich, Oakville ON, 5 µl = 5 µg/sample) were used in the flow cytometry-based cytotoxicity assays (see below). To detect GDTc in mouse blood and tissues, we used anti-CD3 APC (BioLegend, UCHT1, 0.5 µg/ml) and anti-CD45-PE (BioLegend, H130, 0.15 µg/ml). All antibodies were diluted in 20 µl FACS buffer (PBS +1% FBS +2 mM EDTA).

### Cytotoxicity Assays

The flow cytometric cytotoxicity assay was carried out as described [30]. In brief, K562 cells were subject to membrane staining with 3 µM PKH-26 dye (Sigma-Aldrich, Oakville ON) as follows. Cells were mixed with an equivalent volume of 2× PKH-26 stock solution, then incubated 4 min at room temperature with periodic inversion. An equal volume of serum was added, cells were incubated for 1 min to stop the reaction followed by dilution with an equivalent volume of complete medium. Cells were then spun for 10 min at 400×g. Cells were transferred to a fresh tube, washed, resuspended and then plated into 96-well round bottom plates, to which GDTc were added at a 20∶1 effector:target ratio in a final volume of 200 µl in RPMI 1% BSA (GIBCO Canada Ltd, Burlington ON), and 150 U/ml rIL-2 (Chiron, Ville Saint-Laurent, Quebec). Incubation was for 4 hours at 37 degrees, 5% CO_2_. Cells were then stained with Annexin V-FITC (Biovision, Mountain View CA) and 7-AAD (Sigma-Aldrich, Oakville ON) and subjected to flow cytometry-based analyses. Percentages of Annexin V-positive cells at 4 hours were calculated by subtracting the mean percentage of Annexin V-positive target cells at t = 0 from that at t = 4 hours and dividing by the total effector cell number at t = 0. SEM was calculated and statistical analysis performed with Excel software.

### Colony-forming cell (CFC) assay

10,000 autologous PBMCs were incubated with 200,000 GDTc (mean purity  = 97.8%) in 200 µl RPMI 1% BSA and 150 U/ml rIL-2 at 37°C with 5% CO_2_. After four hours, GDTc were depleted as described below. PBMCs were cultured in Methyl Cellulose H443 (Stem Cell Technologies, Vancouver, BC), seeded at a density of 150 000 cells/plate in triplicate, according to the manufacturer's instructions for 2 weeks prior to colony counting.

### GDTc depletion

Prior to plating cells in Methyl Cellulose H443, GDTc were stained and depleted using the TCR γδ isolation kit (Miltenyi Biotec, Auburn CA) as per the manufacturer's instructions. Following cell labeling, cells were run through an LD depletion column and washed three times with MACS buffer. Cells in the flow-through were then enumerated and plated in Methyl Cellulose H443 (Stem Cell Technologies, Vancouver, BC) according to the manufacturer's instructions for 14 days at 37°C with 5% CO_2_.

### Chromium Release Assay

Cr^51^ release assays were performed according to standard protocols [31]. Data are presented as mean % lysis of duplicate or triplicate samples as indicated (±SD).

#### Mice

NOD.Cg-*Prkdcscid Il2rgtm1Wjl*/SzJ (NSG) mice (Jackson Laboratories, Bar Harbor, ME) were maintained at the Ontario Cancer Institute animal facility. 8–12 week old males were irradiated (250 cGy, ^137^Cs) 4–24 hours prior to tail vein injection of the indicated number of EM-2eGFPluc cells in a final volume of 0.2 ml PBS +0.2% BSA. GDTc were injected intravenously or intraperitoneally (IP) at the indicated doses and timepoints. rIL-2 (Proleukin, Novartis Pharmaceuticals, Canada) was administered (100 IU/mouse) IP concordant with the GDTc injections and weekly thereafter where indicated. Leukemia progression/regression was monitored via IVIS® imaging. Mice were evaluated frequently for symptoms of leukemia and all efforts made to minimize suffering; animals were sacrificed at appropriate humane endpoints.

### Preparation of blood and tissues for flow cytometry

Using a heparinized capillary tube, approximately 50 µl of blood was obtained from the saphenous vein of each mouse. 1.5 ml of ACK red blood cell lysis buffer (0.155 M NH_4_Cl, 10 mM KHC0_3_, 0.1 mM Na_2_EDTA in distilled H_2_0) was added and cells were incubated for 10 min at room temperature with frequent vortexing. Cells were centrifuged for 5 min at 400× g, supernatants removed and then the pellets re-suspended in 10 µl mouse IgG (1∶100, Sigma-Aldrich, Oakville ON) in FACS buffer and incubated for 10 min on ice in the dark. Anti-CD3 APC and anti-CD45 PE antibodies were added to final concentrations of 0.5 µg/ml and 0.15 µg/ml, respectively, in 20 µl final volume. Cells were stained for 20 min on ice in the dark, followed by washing with 1 ml FACS buffer, then fixing with 2% paraformaldehyde in FACS buffer. Cells were strained through a fine mesh before flow cytometric acquisition. Organs were removed from the animals and placed in PBS +1% FCS solution on ice. Tissues were homogenized by placing small pieces between mesh squares in PBS +1% FCS and scraping with forceps; aliquots were removed for further processing. ACK lysis and staining were performed as described above.

#### Statistics

Kaplan-Meier survival curves, Logrank tests and student's t tests were performed with GRAPHPAD PRISM 5 for Windows (version 5.03).

## Results

### Gamma delta T cells isolated from human blood can be expanded *in vitro* and have a predominantly effector memory phenotype

**Figure 1:**
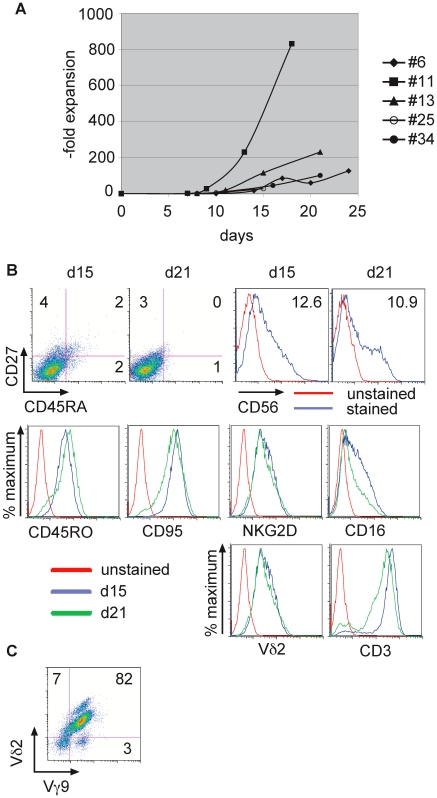
Human Vgamma9 Vdelta2 gamma delta T cells expanded *in vitro* have an effector memory phenotype. a) Fold expansion data for gamma delta T cell cultures derived from a single donor exhibit variable yet high yields. The isolation number (#6, #11, #13, #25, #34) is indicated; cultures shown were derived from the same donor at different times, a minimum of 2 months apart. b) Expanded cells from culture #25 were harvested on days 15 and 21, then stained for the indicated surface markers and subject to flow cytometry. % live cells which stained for CD27 and/or CD45RA are shown on the dot plots. Histogram overlays show the indicated marker expression as % maximum. CD56 expression is displayed for stained and unstained cells on days 15 and 21 as indicated above the respective panels; –fold mean fluorescence intensity over the unstained control is indicated. Histogram overlays show expression of markers CD45RO, CD95, NKG2D, CD16, Vdelta2 TCR and CD3 on day 15 (blue) and day 21 (green), with an unstained sample serving as negative control (red). Shown is a representative example; (n = 8, 2 different donors). c) A representative flow cytometric analysis of expanded gamma delta T cells indicates that Vdelta2 is paired with Vgamma9 (n = 3 different donors). Cells were harvested and stained with anti-Vgamma9 biotin antibody, followed by anti-streptavidin-APC and anti-Vdelta2PE antibodies.

We have developed a protocol to obtain high yields and purity of GDTc isolated from healthy donor-derived peripheral blood mononuclear cells (PBMCs). Expansion yields from a single donor (Donor 1) were variable, ranging from 29-fold to 832-fold (Fig. 1a). %Vdelta2 (Vd2) for these expansions were greater than 74%. Please see Supplementary Table S1 for more information about these cultures, and Supplementary Figures S1, S2, S3, S4 and S5 for supporting flow cytometry data. The majority of the expanded cultures comprised CD27- and CD45RA-negative effector memory cells (Fig. 1b), as defined by Dieli et al [32]. A representative example of flow cytometry performed on days 15 and 21 of culture is shown in Fig. 1b. Examination of histogram overlays shows that expression of all markers decreased only slightly from d15 to d21. For example, the activation marker CD56 was virtually unchanged, from a 12.6-fold mean fluorescence intensity increase over unstained on d15 to a 10.9-fold increase on day 21. Histograms show that the memory marker CD45RO was highly expressed (92% and 87%), as was CD95 (Fas, 99% and 94%). CD16 expression was variable, at 46%+ on d15 to 30%+ on d21. Cells were 92% Vd2+ and 80% NKG2D+ on d15 and similarly 85% Vd2+ and 77% NKG2D+ on day 21. High TCR levels were evidenced by CD3 expression (94% and 89%, respectively). Please see Supplementary Table S2 for percentages and mfi values; Supplementary Figure S6 shows a titration of the anti-CD27 APC antibody. Vd2 cells were all Vgamma9 (Fig. 1c, n = 3 different donors, shown is a representative example).

### Expanded gamma delta T cells are selectively cytotoxic against Ph^+^ leukemia cell lines and not autologous hematopoietic progenitors

**Figure 2:**
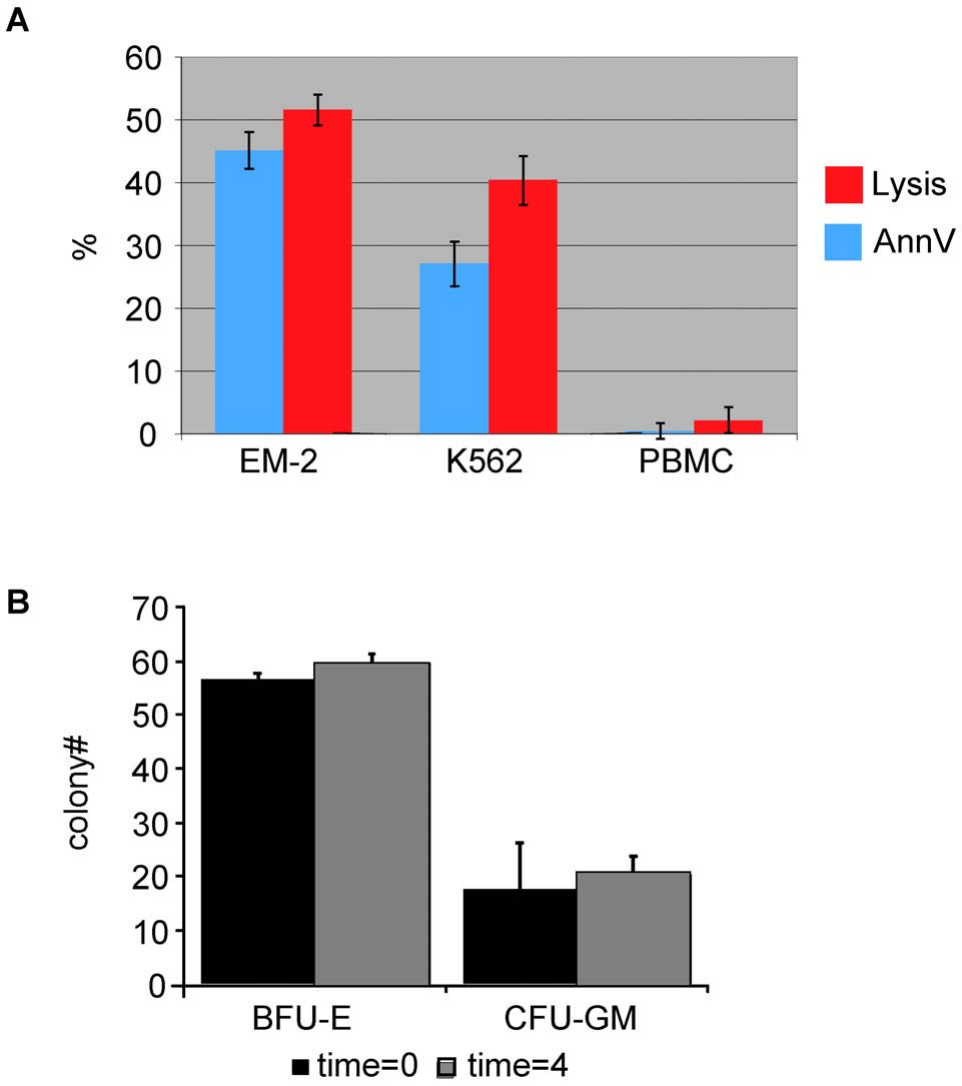
Expanded gamma delta T cells are selectively cytotoxic against Ph^+^ leukemia cells and not autologous hematopoietic progenitors. a) Gamma delta T cells are selectively cytotoxic against Ph^+^ leukemia cell lines K562 and EM-2, but not against normal autologous hematopoietic progenitors, as assessed by a flow cytometric cytotoxicity assay (n = 9 different donors, minimum 6 replicates/donor). Gamma delta T cells were incubated with target cells for 4 hours at an effector:target ratio of 20∶1. Mean % AnnexinV and %lysis ± standard error of target cells is shown. Data were acquired via FACSCalibur and analysed with CellQuest™ software. b) Co-culture of gamma delta T cells with PBMCs does not adversely affect PBMC growth. 200,000 gamma delta T cells were co-incubated with 10,000 autolougous PBMC at 20∶1 effector:target ratio for 0 or 4 hours as indicated. Subsequently, gamma delta T cells were depleted and of the remaining cells, 150,000 per plate were seeded in triplicate for colony-forming assays for normal hematopoietic progenitors, BFU-E and CFU-GM (n = 3 different donors in triplicate).

GDTc isolated from nine healthy donors were expanded *in vitro* and then co-incubated with K562 or EM-2 cells or autologous PBMCs at a 20∶1 effector to target (E:T) ratio for 4 hours. The percentage of apoptotic EM-2 cells was 45% ±3% (mean ± SE) compared to 27% ±2% for K562 cells and only 0.4% ±1.1% for PBMCs as identified by Annexin V staining and flow cytometric analysis (Fig. 2a). Focusing on the live target cell population in this assay, which was both AnnexinV and 7-AAD negative, and applying the formula: % lysis  =  [(%live target_t = 0_ - %live target_t = 4_ hours)/(%live target_t = 0_)] ×100% yielded 52% ±2% EM-2, 40% ±4% K562 and 2% ±2% PBMCs (Fig. 2a). Thus, incubation of autologous PBMC with GDTc did not result in significant lysis of PBMCs, but GDTc are selectively cytotoxic against these Ph^+^ leukemic cell lines.

To further investigate the effects of GDTc against hematopoietic progenitors, GDTc were co-incubated with autologous PBMCs for 4 hours and compared to a 0 hour control. Enumeration of early erythroid (BFU-E) and granulocyte-macrophage colonies (CFU-GM) derived from cells at both time points demonstrated comparable colony numbers (Fig. 2b), verifying that GDTc are not cytotoxic towards the autologous hematopoietic progenitors.

### EM-2eGFPluc cells are fluorescent and bioluminescent

**Figure 3:**
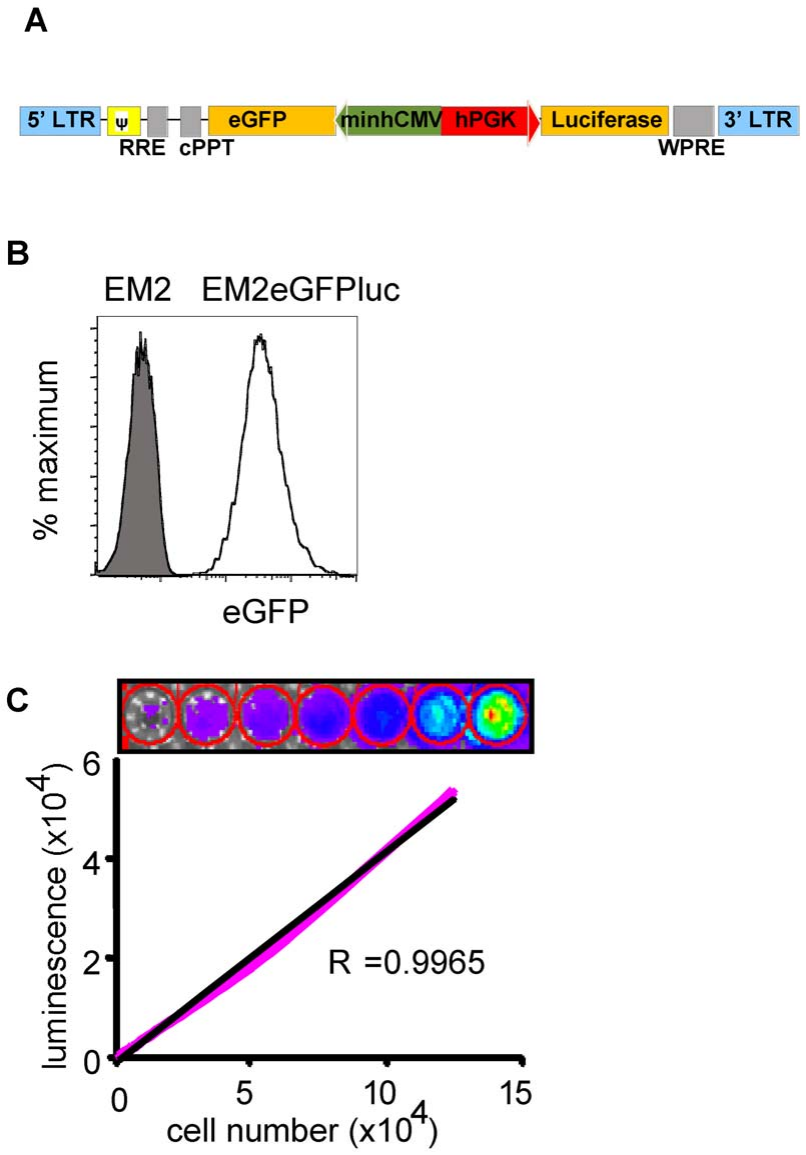
EM-2eGFPluc cells are fluorescent and bioluminescent. a) Scheme of the recombinant lentivector constructed for these studies showing the dual promoter system driving eGFP and Luciferase expression in transduced cells. b) The parental cell line, EM-2, and transduced line, EM-2eGFPluc, were subject to flow cytometry. Shown is a representative example. Cells were assessed by flow cytometry prior to each injection into NSG mice (n = 9). c) EM2eGFPluc cell bioluminescence directly correlates with cell number. Cells were harvested and density adjusted in PBS, then subject to serial dilution 1∶2 from well to well. After addition of luciferin, cells were imaged using IVIS® technology and then quantified using Living Image™ software. Luminescence was plotted against cell number and linear regression calculated using Microsoft Excel software (n = 2).

In order to test GDTc therapy in a pre-clinical setting, we developed a xenogeneic model of Ph^+^ leukemia using fluorescent and bioluminescent leukemia cells. For this, we first transduced EM-2 with a lentiviral vector encoding enhanced green fluorescent protein (eGFP) and luciferase. The recombinant lentivector was constructed such that a dual promoter system drives eGFP and Luciferase expression in transduced cells (Fig. 3a). Flow cytometry was performed on EM-2 and the transduced cells to confirm that EM-2eGFPluc are fluorescent (Fig. 3b). We subjected serially diluted cells to bioluminescent imaging to confirm that EM-2eGFPluc bioluminescence is directly proportional to cell number (Fig. 3c, linear regression  = 0.9965).

Transduction of the parental line did not affect the ability of GDTc to recognize and become activated by these targets (Supplementary Fig. S7). Likewise, transduction of the vector into RAJI cells, which do not activate GDTs, did not result in CD107 expression of GDTc (Supplementary Fig. S8).

### A novel xenogeneic bioluminescent Ph^+^ leukemia model

**Figure 4:**
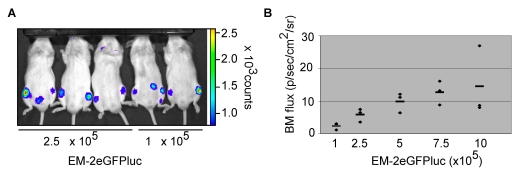
EM-2eGFPluc engraft the bone marrow in a novel *in vivo* xenogeneic Ph^+^ leukemia model. a) NSG mice were injected with EM-2eGFPluc iv on day 0. On day 21, mice were injected with luciferin, anesthetized and imaged using IVIS® technology. Shown here are three mice that received 2.5×10^5^ and two mice that were injected with 1×10^5^ EM-2eGFPluc on day 0. In total, 17 mice were used in this experiment: 3 per group at doses of 1, 2.5, 5, 7.5 and 10×10^5^ EM-2eGFPluc; 1 mouse was injected with 0.4×10^5^ EM-2eGFPluc; 1 mouse was injected with PBS +0.2% BSA alone. b) On day 28, mice were injected with luciferin, anesthetized and imaged using IVIS® technology. Bioluminescence was quantified using Living Image™ software and bone marrow flux per mouse plotted against injected EM-2eGFPluc. Shown here are the results from the 5 groups of 3 mice described in Fig. 4a. Bone marrow (BM) flux units are photons/second/square centimeters/steradian.

We have established a xenogeneic mouse model of Ph^+^ leukemia by injecting EM-2eGFPluc cells intravenously (iv) into non-lethally irradiated NOD.Cg-*Prkdcscid Il2rgtm1Wjl*/SzJ (NSG) mice. To assess the dose of EM-2eGFPluc required to elicit consistent bone marrow engraftment, we irradiated 8.5-week old mice with 2.5 Gy one day prior to leukemia cell injection. We then injected 1–10×10^5^ EM-2eGFPluc cells iv (3 mice at each dose of 1, 2.5, 5, 7.5 and 10×10^5^ plus 1 PBS control) and monitored engraftment via *in vivo* (IVIS) imaging at 21, 28 and 32 days post-injection. On d21, 5 minute scans showed luciferase signals corresponding to bone marrow engraftment. Shown here is a representative scan of 3 mice that received 2.5 and 2 mice with 1×10^5^ EM-2eGFPluc (Fig. 4a). Consistent engraftment of leukemia cells in the bone marrow of both hind limbs was evident even in mice injected with the lowest EM-2eGFPluc dose of 1×10^5^ cells. Bone marrow signals from scans performed on d28 were quantified using Living Image™ software and plotted against injected cell number, revealing that EM-2eGFPluc engrafted bone marrow in a dose-dependent manner (Fig. 4b).

### Gamma delta T cells persist and remain viable in our xenogeneic leukemia model

**Figure 5:**
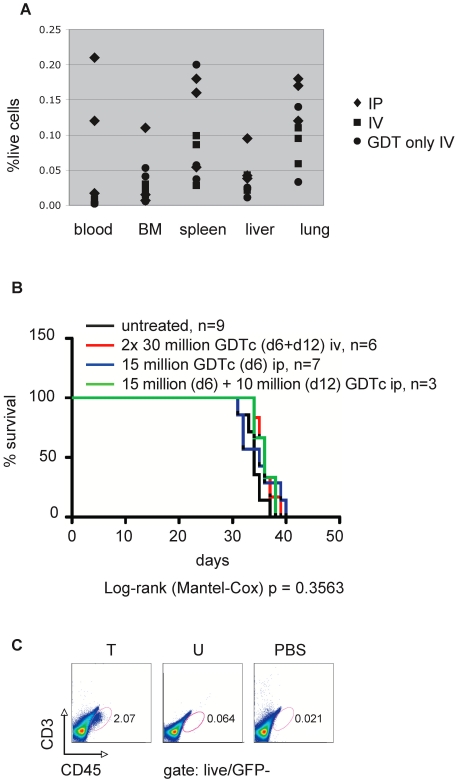
Higher gamma delta T cell levels result when they are injected intraperitoneally versus intravenously. a) Irradiated NSG mice were injected with 0 (n = 3 mice GDT only iv) or 1.5 million EM-2eGFPluc (n = 6 mice) on day 0. On day 6, 3 mice were injected with 30 million Donor 2 GDTc intraperitoneally (IP); 6 mice were injected iv (IV+GDTonlyIV). Donor 2 GDTc had been cultured for 20 days prior to injection. Mice were sacrificed on day 20, the indicated tissues were prepared for flow cytometric assessment. Gamma delta T cells were stained with anti-CD3 APC and anti-CD45 PE antibodies. b) 1.5 million EM-2eGFPluc cells were injected intravenously into irradiated NSG mice (n = 25). 30 million Donor 1 GDTc (Isolation 13, days 15 and 21) were injected iv on days 6 and 12 post-leukemia cell injection, along with 100 IU recombinant human IL-2/mouse, plus one additional IL-2 dose on day 21 (n = 6, red line). 15 million GDTc (Isolation 25, day 15) were administered ip on day 6 (n = 10, blue and green lines); a second dose of 10 million (Isolation 25, day 21) was given on day 12 (n = 3, green line). IL-2 was administered on GDTc injection days only (green and blue). Survival curves were not significantly different (Log-rank/Mantel-Cox p = 0.3563). c) Bone marrow from treated (T), untreated (U) and PBS control mice was extracted and stained with anti-CD3APC and anti-CD45PE antibodies and then subject to flow cytometric analysis. A live cell gate was made based on forward and side scatter properties; the GFP-negative population of live cells is shown here. This is a representative example of bone marrow samples taken at endpoint from the experiment described in b (T single dose, blue line; U, black line). Bone marrow samples were obtained from 2 PBS controls, 1 untreated and 7 treated mice in total.

We then investigated the viability and biodistribution of human GDTc effector cells in this model. We initially injected GDTc iv and were able to detect these cells in the mice until the experimental endpoint (data not shown); however, we wanted to see whether we could further enhance levels by injecting GDTs intraperitoneally (ip). Indeed, we found more ip-injected than iv-injected GDTs in our established leukemia model (Fig. 5a). We irradiated mice in the evening prior to injection day and then injected six animals with 1.5×10^6^ EM-2eGFPluc cells iv and three animals with PBS. Six days later, 2 groups of 3 tumour-bearing mice each were injected with 30×10^6^ expanded GDTc from Donor 2 either iv or ip. The remaining three mice were injected with GDT iv and constituted the “GDT only” group. Mice were sacrificed on day 20; blood and tissues were processed for flow cytometry. The data show more ip-injected than iv-injected GDTc in all tissues analyzed (Fig. 5a). It is noteworthy that none of the mice receiving 30×10^6^ GDTc developed graft-versus-host disease (GvHD), proving that at least one dose of large numbers of human GDTc were well tolerated in NSG mice.

In early therapy experiments, we had injected 1.5×10^6^ EM-2eGFPluc cells iv into NSG mice to establish leukemia and then administered GDTc therapy at various time points and doses, in an attempt to halt leukemia progression. In one such experiment, 30×10^6^ Donor 1 GDTc were injected iv on days 6 and 12 post-leukemia cell injection, along with 100 IU recombinant human IL-2/mouse, plus one additional IL-2 dose on day 21 (Fig. 5b). In the next experiment using the same initial leukemia dose, but with ip GDTc injections, Donor 1 GDTc expansion was not as great; 15×10^6^ GDTc were administered ip in 10 mice on day 6 followed by a second dose of 10×10^6^ in only 3 mice (Fig. 5b). IL-2 was administered on GDTc injection days only. The Kaplan-Meier survival curves show that under these conditions in this model, GDTc therapy provided no survival advantage (Logrank/Mantel-Cox, p = 0.3563), whether GDTc were injected ip or iv. However, we were able to detect GDTc in the bone marrow of infused mice at necropsy (Fig. 5c) indicating that GDTc reached the primary site of leukemia cell engraftment. For additional flow cytometry data from this experiment, please see Supplementary Figure S9.

**Figure 6:**
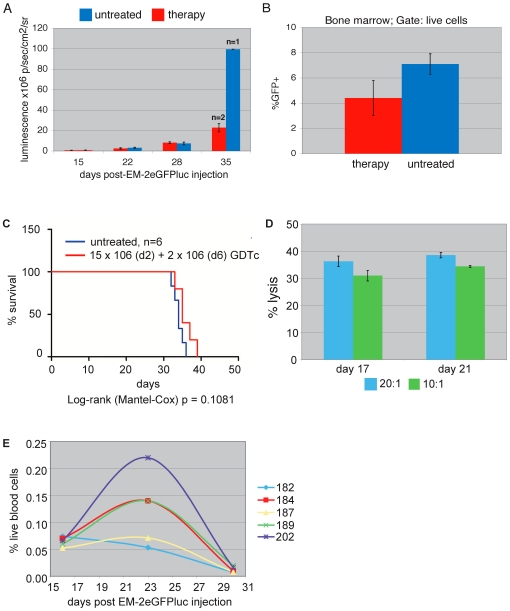
Gamma delta T cell therapy lowers bone marrow leukemia burden. a) 1 million EM-2eGFPluc cells were injected intravenously into NSG mice (n = 11). 15 million Donor 1 GDTc were injected intraperitoneally on day 2 and 2 million on day 6 post-leukemia cell injection (n = 5). All mice received 100 IU recombinant human IL-2 intraperitoneally on gamma delta T cell injection days and then weekly thereafter. IVIS® imaging was performed on the indicated days and bone marrow bioluminescence was quantified using Living Image™ software. Untreated (blue) n = 6 and therapy (red) n = 5, except on day 35, where untreated n = 1 and treated n = 2, since most mice were sacrificed between days 28 and 35. Shown are mean luminescence ± standard error. b) At experimental endpoint, bone marrow was extracted and GFP positive cells detected by flow cytometry. Results from therapy mice are shown in red and untreated in blue (error bars represent standard error; n = 5 and 5, respectively). c) Kaplan Meier survival curve for the experiment described in a) and b), with no significant survival advantage achieved (untreated blue line (n = 6) treated red (n = 5); Log-rank/Mantel-Cox, p = 0.1081). d) Donor 1 gamma delta T cells were tested in Cr^51^ release assays against EM-2eGFPluc targets on injection days for the experiment shown in a)-c). Effectors and Cr^51^-labeled targets were incubated at 20∶1 (blue) and 10∶1 (red) for 4 hours at 37°C and Cr^51^ release measured (n = 1 donor in triplicate). Shown is the mean ± standard deviation. These results are representative for gamma delta T cell cytotoxicity measured on injection days in other therapy experiments (n = 4 independent experiments in tripicate, 2 different donors). e) For the experiment shown in a)-d), weekly blood samples were taken via saphenous vein and blood was stained with anti-human CD3 and anti-human CD45 antibodies to detect gamma delta T cells on the indicated days. Gating was done on the live cell population in forward and side scatter. Individual treated mice are indicated by number (n = 5 treated mice in this experiment). Monitoring of gamma delta T cells in blood was performed in every therapy experiment described (n = 4 independent *in vivo* experiments).

For the next experiment, we used a lower initial leukemia cell dose (1×10^6^ EM-2eGFPluc, n = 11) and earlier GDTc treatments, with 15×10^6^ cells injected ip on day 2 and a further 2×10^6^ ip given on day 6 (n = 5, Fig. 6). In this experiment, IL-2 was administered ip with GDTc injections and then weekly thereafter to promote survival of injected GDTc.

We monitored bone marrow bioluminescence over time (Fig. 6a). In this experiment, bioluminescence between treated and untreated mice was similar at early time points, in contrast to previous experiments (data not shown). However, of the three survivors on d35, the treated mice had much lower signals than untreated, suggesting that GDTc resulted in a lower leukemia burden, hence longer survival. Flow cytometry data at the experimental endpoint also showed a decreased eGFP+ cell population in the two treated mice versus untreated animals (0% and 2.8% compared to 5.5%, respectively). Overall, the endpoint bone marrow data ranged considerably in the treated group (0% –8.0%, mean 4.4% ±3.1), rendering the difference between treated and untreated groups insignificant (student's t-test, p = 0.13); in contrast, untreated mice exhibited a highly consistent leukemia burden 7.1% ±1.4, suggesting that GDTc therapy did indeed have some influence in the bone marrow (Fig. 6b). However, no significant survival advantage was achieved (Fig. 6c, Logrank/Mantel-Cox, p = 0.1081).

To ensure that the injected GDTc were indeed functional, we assessed the ability of EM-2eGFPluc targets to activate the cells on injection days with the CD107 assay (data not shown). Cr^51^ release assays also confirmed GDTc cytotoxicity against EM-2eGFPluc targets. Shown in Fig. 6d are Cr^51^ data for this particular experiment. Percent lysis at 20∶1 (blue) and 10∶1 (red) E:T ratios were 36.2% ±1.9 (mean ± SD) and 31.0% ±2.0, respectively, on culture day 17, and slightly higher at 38.5% ±1.0 and 34.4±0.4% on day 21, confirming GDTc cytotoxicity against EM-2eGFPluc targets.

GDTc survived in the mice until the experimental endpoint, up to 33 days after administration of the second GDTc dose. Weekly blood samples were taken via saphenous vein on days 16, 23 and 30 post-leukemia cell injection. Blood was stained with anti-human CD3 and anti-human CD45 antibodies to detect the presence of GDTc (Fig. 6e). In 4 of 5 mice, GDTc numbers increased dramatically by day 23; the mean was 0.13% ±0.06 (± SD) of live cells compared to 0.06% ±0.01 on day 16, but decreased again by day 30, to an average of 0.01% ±0.01. At endpoint, between days 33 and 39, GDTc were found in the blood of all five therapy mice (0.25% ±0.19, n = 5); since these samples were obtained from post-necropsy heart puncture, they could not be compared directly to previous samples that had been obtained via saphenous vein of live mice. EM-2eGFPluc blood levels were not significantly lower in the treated group (data not shown, 0.24% ±0.24 SD in treated versus 0.31% ±0.33 SD in untreated mice).

While GDTc could not be detected in the bone marrow of all but one mouse (#202), they were found in the spleen (0.99% ±2.11, n = 5). Mouse #202 had exceptionally high GDTc levels at necropsy (Supplementary Figure S10). Our data show that human GDTc are able to survive at least 39 days in this mouse model.

## Discussion

Several protocols are now available to expand GDTc *in vitro*; most rely on synthetic phosphoantigens such as bromohydrin pyrophosphate (IPH1101, formerly Phosphostim™) or aminobisphosphonates like Zoledronate that preferentially expand the Vγ9Vδ2 subset in the context of whole PBMCs [33], [34]. The purity of GDTc obtained using these protocols is variable; however, this can be improved by GDTc sorting and culturing with irradiated PBMC feeders in the presence of mitogen [35]. A recently published protocol depletes PBMCs of CD4+ and CD8+ cells and, using anti-CD3 stimulation, subsequently expands Vdelta1 and/or Vdelta2 GDTc, in proportion to their ratio in peripheral blood [36]. In contrast, our protocol requires antibodies only for the initial selection, but not for the further expansion, of GDTc. Feeder cells are not required; these complicate a clinical protocol by introducing extra handling, thereby increasing workload and the potential for contamination. With our protocol, we can expand GDTc to clinically relevant numbers; in many cases, we can even drive expansion of the Vdelta1 or Vdelta2 subset irrespective of their proportion in donor blood (Siegers et al, in press).

It will be important to establish whether it is possible to expand patient GDTc with our protocol. Indeed, in the clinical setting, it would be best to subject patient samples to a preliminary expansion test in order to determine their responsiveness to this protocol and thereby gauge the feasibility of generating clinically relevant numbers of GDTc. To this end, it would also be advisable to screen each patient's GDTc against a panel of human AB sera at the same time, to determine the optimum serum for clinical expansion.

The majority of GDTc generated using our protocol are effector memory cells, CD45RA^−^, CD45RO^+^ [32], [37] and CD27^−^ [32]. This is in accordance with GDTc expanded from imatinib-treated CML patients, which are predominantly central memory (CD45RA-CD27+) in peripheral blood, yet after expansion with bromohydrin pyrophosphate attain the CD45RA-CD27- effector memory phenotype (Helene Sicard, personal communication, unpublished data). In contrast, in a recently published study, an increase in late memory cytotoxic lymphocytes was found in dasatinib-treated patients, as well as an overall increase in GDTc levels compared to diagnosis [38], adding to a growing body of evidence suggesting that individual TKIs have differing immunomodulatory properties in the context of CML [25].

In our cultures, high CD95^+^ expression may indicate readiness to undergo *in vivo* deletion [39]. We also observed high levels of NKG2D and moderate CD56 and CD16 expression, all of which are correlated with GDTc cytotoxicity [40], [41], [42]. Importantly, cytotoxicity assays performed on injection days confirmed that these *in vitro*-expanded GDTc were potent killers of EM-2eGFPluc target cells.

As an important step towards validating our pre-clinical model for cell therapy, we have demonstrated that *in vitro*-expanded GDTc are specifically cytotoxic towards Ph^+^ leukemia cells, but not normal autologous peripheral blood cells or hematopoietic progenitor cells.

Our rationale for developing a xenogeneic leukemia model, as opposed to a murine model, to test GDTc therapy is because the human Vγ9Vδ2 GDTc subset, which is cytotoxic to Ph^+^ leukemia cells, does not exist in mice [43], [44]. We chose the NSG strain since engraftment of human hematopoietic cells in these mice is superior compared to NODscid [45]. Even so, irradiation of these mice prior to leukemia cell injection is needed, as one experiment performed in the absence of irradiation resulted in lesser and inconsistent leukemia cell engraftment (data not shown). The use of bioluminescent leukemia cells in this model provides several advantages: 1) leukemia progression and regression can be easily monitored; 2) leukemia burden can be quantified; and 3) less animals are required because it is not necessary to sacrifice animals at several time points to monitor disease. We chose to use EM-2eGFPluc cells for this model because their engraftment is mainly localized to the bone marrow, the site of hematopoeisis. Earlier attempts using K562eGFPluc cells resulted in an unsatisfactory pathology, with inconsistent tumour cell engraftment of other anatomical locations and not bone marrow (data not shown), similar to a recently published xenograft model using MM1 leukemic cells [46]. Importantly, in that study, D'Asaro and colleagues show impressive killing of zoledronate-pretreated IM-sensitive and -resistant CML cells by patient-derived Vdelta2 GDTc *in vitro* and *in vivo*. Though combination treatment of GDTc, zoledronate and IL-2 in their xenograft model eradicated bioluminescence signals and greatly improved survival, the *in vivo* model pathology in that case did not resemble CML. [46]. Thus, they were unable to address whether GDTc migrate to bone marrow and provide defense at this critical anatomical location. Likewise, in xenograft experiments performed by a different group, subcutaneous injection of lung cancer cells and GDTc provided significant survival advantages to treated mice, but the model did not emulate human disease [36].

In early stages of our model system, leukemia engraftment closely recapitulated human chronic phase CML; however, at later points, aggressive disease resembled blast crisis, at which point GDTc likely became overwhelmed, possibly accounting for the lack of survival advantage in treated mice. Our model could be potentially improved by administration of IM, to better emulate the situation in the majority of CML patients. Use of GDTc plus aminobisphosphanates such as zoledronate or aledronate may have dramatically improved outcomes, as observed in other experimental systems [46], [47]. A drawback to our system was that GDTc were injected in the absence of other human immune cells that could play an important role in augmenting antitumor cytotoxicity and GDTc survival; thus, this model may underestimate GDTc potential.

At best, we could administer only two doses of GDTc therapy due to ethics board restrictions allowing us only one blood draw every two months from the same donor. Increased frequency of access to blood from the same donor would have allowed for more therapy doses and possibly improved our chances of obtaining a significant response. Indeed, Kabelitz and colleagues saw significantly improved survival with increasing frequency of GDTc therapy in both their MeWo melanoma and PancTu1 pancreatic adenocarcinoma models [47]. They were able to generate multiple GDTc doses over time by using re-stimulation and irradiated feeder cells. Our protocol is much less involved, but unfortunately, we are unable to recover frozen and thawed polyclonal GDTc batches to use at will. Overcoming this obstacle would provide a great advantage to the GDTc field at large.

Despite these limitations, a measure of success was achieved with GDTc therapy as evidenced by the decreased leukemia burden in the bone marrow of some treated mice. Indeed, bone marrow leukemia was not necessarily responsible for the demise of mice in these experiments; rather, migration of leukemia cells to the central nervous system late in the experiments caused paralysis in most, at which point the mice were sacrificed on humane grounds. Again, IM alone or in combination with GDTc activators might prevent this migration and improve the model as well as conditions for therapy.

We are the first to formally document long-term survival of GDTc in the bone marrow in a xenograft setting. This is significant, as primary GDTc longevity *in vitro* is limited to 14–21 days, depending on the protocol. In our therapy experiments, GDTc were cultured 16–21 days before injection and were still found alive in the mice up to 33 days later. This is similar to results obtained by Kabelitz and colleagues, who injected multiple doses of GDTc clones and found small numbers remaining in peritoneal exudates and spleen cells in SCID mice up to 30 days post-injection [47]. Indeed, we were very encouraged to find GDTc in the bone marrow at endpoint in most of our experiments. Our inability to detect GDTc in some cases might have been partly due to sample preparation, since unpredictable endpoints sometimes led to sample fixation for various lengths of time. Also the time lapse between the final GDTc injection and experimental endpoint likely played a role. GDTc were detectable in bone marrow when the second GDTc dose was on day 12 (Fig. 5B, red line), yet not consistently in an experiment where GDTc were last administered on day 6 (Fig. 6). Importantly, we found no evidence of graft-versus-host disease in these mice, as high numbers of human GDTc, up to two doses of 30 million per dose, were well tolerated. However, this may become an issue with increasing numbers and frequency of doses, as Kabelitz and colleagues observed beyond five doses of 10 million GDTc in their mouse models [47].

In summary, we have devised a protocol to expand GDTc to clinically relevant numbers, developed a bioluminescent xenogeneic model of Ph^+^ leukemia and tested GDTc therapy in this model. Although no significant survival advantage was achieved under our conditions, GDTc therapy was well tolerated and GDTc survived for extended periods in the mice. GDTc were often found in bone marrow and therapy decreased leukemia burden in some treated mice, showing that GDTc migrate to, and are active at the site of leukemia. We are currently optimizing treatment regimens and further exploring GDTc cytotoxicity mechanisms and migration in this model.

## Supporting Information

Table S1 **Purity, passaging and viability of gamma delta T cell cultures derived from Donor 1.** Gamma delta T cells were isolated from peripheral blood of Donor 1 and cultured as described in Materials and Methods. d  =  day; bkg adj  =  background adjusted (unstained control values were subtracted from those of stained samples); GDT  =  gamma delta T cell antigen receptor positive cells; Vd1  =  Vdelta1 and Vd2  =  Vdelta2 are indicated for cultures that were not stained with anti-GDT antibody; AB  =  alpha beta T cell antigen receptor positive cells; * days of initial exposure to Concanavalin A; **Calculated from %CD3 – %AB, since GD TCR staining did not work; -fold exp (d total)  =  -fold expansion (total number of days in culture). Viability was calculated: (live/(live+dead)) x 100%. Live and dead cells were distinguished via Trypan Blue exclusion.
(DOC)

Table S2 **Flow cytometry values showing percent positive and corresponding mean fluorescent intensity for various surface markers.** Gamma delta T cell cultures were harvested and stained for the indicated surface markers on days 15 and 21 of culture. d  =  day; mfi  =  mean fluorescence intensity; Vd2  =  Vdelta2 T cell antigen receptor. Table values are for flow cytometric data shown in Fig. 1B.
(DOC)

Figure S1 **Supporting flow cytometry data for gamma delta T cell culture derived from Donor 1 isolation#6.** d  =  culture day; GD TCR  =  gamma delta T cell antigen receptor; AB TCR  =  alpha beta T cell antigen receptor. The upper panels show purity of gamma delta T cells isolated via positive selection (MACS magnetic sorting, Miltenyi). The lower panels show staining of cells harvested on culture day 17. Live lymphocytes were gated in forward and side scatter (not shown). Negative controls in red are the unstained peripheral blood mononuclear cell fraction before sorting (preMACS, top panels) or unstained cells (bottom left). On the upper left, gamma delta T cells are bound to beads that are FITC labeled. This fraction was also labeled with anti-AB TCR antibody (top right panel). Percent positive GD TCR and AB TCR and histogram gate are indicated. The lower right panel is a dot blot indicating the percentage of AB TCR positive cells in culture on day 17.
(TIFF)

Figure S2 **Supporting flow cytometry data for gamma delta T cell culture derived from Donor 1 isolation#11.** d  =  culture day; GD TCR  =  gamma delta T cell antigen receptor; AB TCR  =  alpha beta T cell antigen receptor. The upper panels show purity of gamma delta T cells isolated via positive selection (MACS magnetic sorting, Miltenyi). On the upper left, gamma delta T cells are bound to beads that are FITC labeled. This fraction was also labeled with anti-AB TCR antibody (top right panel). Gating and percent positive GD TCR and AB TCR are indicated. The middle and lower panels show staining of cells harvested on culture days 12 and 18, respectively. Live cells were gated in forward and side scatter (not shown). Negative controls in red are the unstained peripheral blood mononuclear cell fraction before sorting (preMACS, top panels) or unstained cells (middle and bottom left).
(TIFF)

Figure S3 **Supporting flow cytometry data for gamma delta T cell culture derived from Donor 1 isolation#13.** d  =  culture day; GD TCR  =  gamma delta T cell antigen receptor; AB TCR  =  alpha beta T cell antigen receptor; Vdelta2  =  Vdelta2 GD TCR. The upper panels show purity of gamma delta T cells isolated via positive selection (MACS magnetic sorting, Miltenyi). On the upper left, gamma delta T cells are bound to beads that are FITC labeled. This fraction was also labeled with anti-AB TCR antibody (top right panel). Gating and percentage of cells positive for the indicated receptors are shown. The middle and lower panels show staining of cells harvested on culture days 8, 15 and 21, respectively. Live cells were gated in forward and side scatter (not shown). Negative controls in red are the unstained peripheral blood mononuclear cell fraction before sorting (preMACS, top left panel), unstained MACS positive fraction unstained (MACS+, top right) or unstained cells (all other panels).
(TIFF)

Figure S4 **Supporting flow cytometry data for gamma delta T cell culture derived from Donor 1 isolation#25.** d  =  culture day; GD TCR  =  gamma delta T cell antigen receptor; AB TCR  =  alpha beta T cell antigen receptor; Vdelta1  =  Vdelta1 GD TCR; Vdelta2  =  Vdelta2 GD TCR. The upper panels show purity of gamma delta T cells isolated via positive selection (MACS magnetic sorting, Miltenyi). On the upper left, gamma delta T cells are bound to beads that are FITC labeled. This fraction was also labeled with anti-AB TCR antibody (top right panel). Gating and percentage of cells positive for the indicated receptors are shown. The middle and lower panels show staining of cells harvested on culture days 10, 15 and 21, respectively. Live cells were gated in forward and side scatter (not shown). Negative controls in red are the unstained peripheral blood mononuclear cell fraction before sorting (preMACS, top panel) or unstained cells (all other panels).
(TIFF)

Figure S5 **Supporting flow cytometry data for gamma delta T cell culture derived from Donor 1 isolation#34.** d  =  culture day; GD TCR  =  gamma delta T cell antigen receptor; AB TCR  =  alpha beta T cell antigen receptor; Vdelta1  =  Vdelta1 GD TCR; Vdelta2  =  Vdelta2 GD TCR. The upper panels show purity of gamma delta T cells isolated via positive selection (MACS magnetic sorting, Miltenyi). On the upper left, gamma delta T cells are bound to beads that are FITC labeled. This fraction was also labeled with anti-AB TCR antibody (top right panel). Gating and percentage of cells positive for the indicated receptors are shown. The middle and lower panels show staining of cells harvested on culture days 9 and 16, respectively. Live cells were gated in forward and side scatter (not shown). Negative controls in red are the unstained peripheral blood mononuclear cell fraction before sorting (preMACS, top left panel) or unstained cells (bottom left panel).
(TIFF)

Figure S6 **Titration of anti-CD27 APC antibody.** Aliquots of the MACS negative fraction (9 x 10^5^ cells/aliquot) were stained with the indicated dilutions of anti-CD27 APC antibody in 20 µl for 20 min, washed and subject to flow cytometry. The negative control (red line) is unstained MACS negative cells. The upper panel shows histogram overlays of all dilutions tested. Mean fluorescence intensity (mfi) values are indicated. The lower panel shows overlays of the unstained control (red) and the 1:100 dilution used in Figure 1b (blue).
(TIFF)

Figure S7 **Transduced and parental leukemia cell lines elicit similar CD107 mobilization in gamma delta T cells.** CD107 experiments were performed using Donor 3 gamma delta T cells and target lines EM-2 and EM-2eGFPluc. All samples had been stained using anti-human CD107aAlexa647. Live GDTc were gated; shown is a representative example, n = 3 different donors.
(TIFF)

Figure S8 **Transduced RAJI cells do not activate gamma delta T cells.** CD107 experiments were performed using Donor 2 gamma delta T cells and target lines EM-2, RAJI and RAJIeGFPluc. All samples had been stained using anti-human CD107aAlexa647 and anti-Vdelta2PE antibodies. Cells were gated on live Vdelta2+ GDTc; mean fluorescence intensity values are shown in red and %CD107 in black.
(TIFF)

Figure S9 **Additional bone marrow flow cytometry data from therapy experiment shown in** **Fig. 5c** **.** Bone marrow from treated (T, n  =  7), untreated (U, n  =  1) and PBS control (n  =  2) mice was extracted and stained with anti-CD3APC and anti-CD45PE antibodies and then subject to flow cytometric analysis. The live cell gate was based on forward and side scatter properties; the GFP-negative population of live cells is shown. The percentage of CD3/CD45 positive cells is indicated. * mice that received two gamma delta T cell doses.
(TIFF)

Figure S10 **Gamma delta T cells are found in spleen, blood and bone marrow.** Flow cytometric analysis showing gamma delta T cell engraftment in the tissues of one therapy mouse (#202) at endpoint in the experiment shown in Figure 6. One untreated mouse (#199) is shown as a negative control. Gates and percentages are indicated.
(TIFF)
