# Supplementary material for: Anti-Leukemia Activity of In Vitro-Expanded Human Gamma Delta T Cells in a Xenogeneic Ph+ Leukemia Model
Source: PLoS One. 2011 Feb 3;6(2):e16700. doi: 10.1371/journal.pone.0016700 (PMC3033392; doi:10.1371/journal.pone.0016700)
Supplement: Table S1 — Purity, passaging and viability of gamma delta T cell cultures derived from Donor 1. Gamma delta T cells were isolated from peripheral blood of Donor 1 and cultured as described in Materials and Methods. d = day; bkg adj = background adjusted (unstained control values were subtracted from those of stained samples); GDT = gamma delta T cell antigen receptor positive cells; Vd1 = Vdelta1 and Vd2 = Vdelta2 are indicated for cultures that were not stained with anti-GDT antibody; AB = alpha beta T cell antigen receptor positive cells; * days of initial exposure to Concanavalin A; **Calculated from %CD3 – %AB, since GD TCR staining did not work; -fold exp (d total) = -fold expansion (total number of days in culture). Viability was calculated: (live/(live+dead)) x 100%. Live and dead cells were distinguished via Trypan Blue exclusion. (DOC) [file pone.0016700.s001.doc]

| # | d0 %  (bkg adj) | | % GDT(d) | d passaged | -fold exp  (d total) | pre-injection (bkg adj) | | | |
| --- | --- | --- | --- | --- | --- | --- | --- | --- | --- |
|  | GDT | AB |  |  |  | d | Via-bility | %GDT | %AB |
| 6 | 95.6 | 4.4 | n.d. | 7*,10,14,17,20 | 127 (24) | 17 | 88% | 95.5 | 0.8 |
| 11 | 91.8 | 6.1 | 94.3 (12) | 7*, 9,13 | 832 (18) | 18 | 84% | 98.3 | 1.6 |
| 13 | 97.6 | 1.0 | 87.4** (8) | 8*,11,15 | 232 (21) | 15 | 92% | 84.1 | 3.0 |
|  |  |  |  |  |  | 21 | 82% | 87.9 | 2.6 |
| 25 | 97.2 | 1.2 | 13.0 Vd1,  71.9 Vd2 (10) | 8*,10,15 | 29 (15) | 15 | 80% | 8.2 Vd1,  91.0 Vd2 | 2.7 |
|  |  |  |  |  |  | 21 | 71% | 20.6 Vd1,  74.9 Vd2 | 4.2 |
| 34 | 98.0 | 0.4 | 47.8 Vd1,  16.8 Vd2 (9) | 8*,11,16 | 102 (21) | 16 | 81% | 16.5 Vd1,  80.7 Vd2 | 0.7 |

**S1.**
