# Supplementary material for: Anti-Leukemia Activity of In Vitro-Expanded Human Gamma Delta T Cells in a Xenogeneic Ph+ Leukemia Model
Source: PLoS One. 2011 Feb 3;6(2):e16700. doi: 10.1371/journal.pone.0016700 (PMC3033392; doi:10.1371/journal.pone.0016700)
Supplement: Table S2 — Flow cytometry values showing percent positive and corresponding mean fluorescent intensity for various surface markers. Gamma delta T cell cultures were harvested and stained for the indicated surface markers on days 15 and 21 of culture. d = day; mfi = mean fluorescence intensity; Vd2 = Vdelta2 T cell antigen receptor. Table values are for flow cytometric data shown in Fig. 1B. (DOC) [file pone.0016700.s002.doc]

|  | % positive | | mfi | |
| --- | --- | --- | --- | --- |
| marker | d15 | d21 | d15 | d21 |
| CD56 | 39 | 32 | 6.0 | 4.0 |
| CD45RO | 92 | 87 | 4.9 | 6.8 |
| CD95 | 99 | 94 | 15.8 | 13.2 |
| NKG2D | 80 | 77 | 4.8 | 4.8 |
| CD16 | 46 | 30 | 2.8 | 2.0 |
| Vd2 | 92 | 85 | 21.7 | 11.7 |
| CD3 | 94 | 89 | 45.6 | 25.4 |

**S2.**
