# Supplementary figures and images for: Anti-Leukemia Activity of In Vitro-Expanded Human Gamma Delta T Cells in a Xenogeneic Ph+ Leukemia Model
Source: PLoS One. 2011 Feb 3;6(2):e16700. doi: 10.1371/journal.pone.0016700 (PMC3033392; doi:10.1371/journal.pone.0016700)

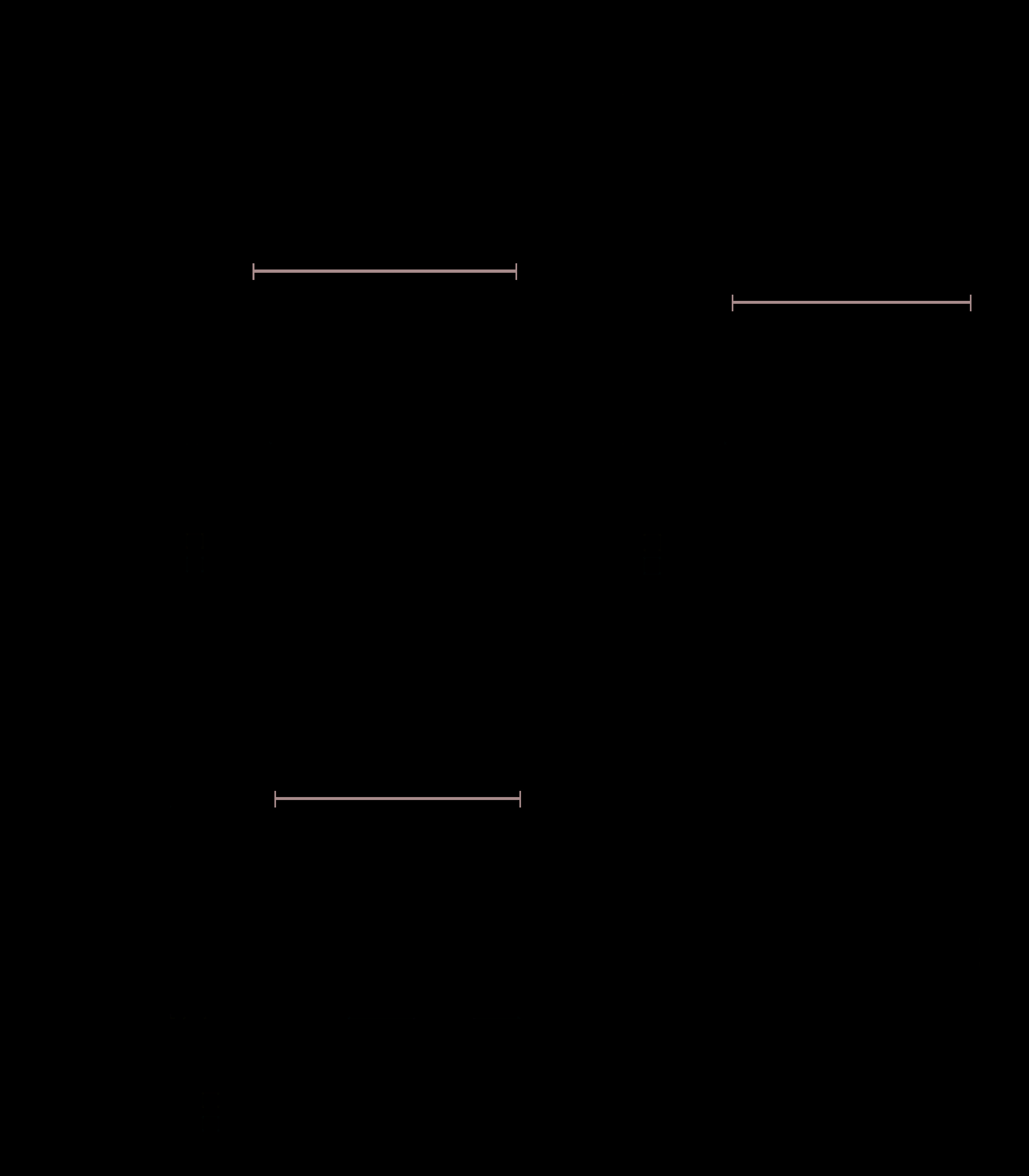

Supplement: Figure S1 — Supporting flow cytometry data for gamma delta T cell culture derived from Donor 1 isolation#6. d = culture day; GD TCR = gamma delta T cell antigen receptor; AB TCR = alpha beta T cell antigen receptor. The upper panels show purity of gamma delta T cells isolated via positive selection (MACS magnetic sorting, Miltenyi). The lower panels show staining of cells harvested on culture day 17. Live lymphocytes were gated in forward and side scatter (not shown). Negative controls in red are the unstained peripheral blood mononuclear cell fraction before sorting (preMACS, top panels) or unstained cells (bottom left). On the upper left, gamma delta T cells are bound to beads that are FITC labeled. This fraction was also labeled with anti-AB TCR antibody (top right panel). Percent positive GD TCR and AB TCR and histogram gate are indicated. The lower right panel is a dot blot indicating the percentage of AB TCR positive cells in culture on day 17. (TIFF) [file pone.0016700.s003.tiff]

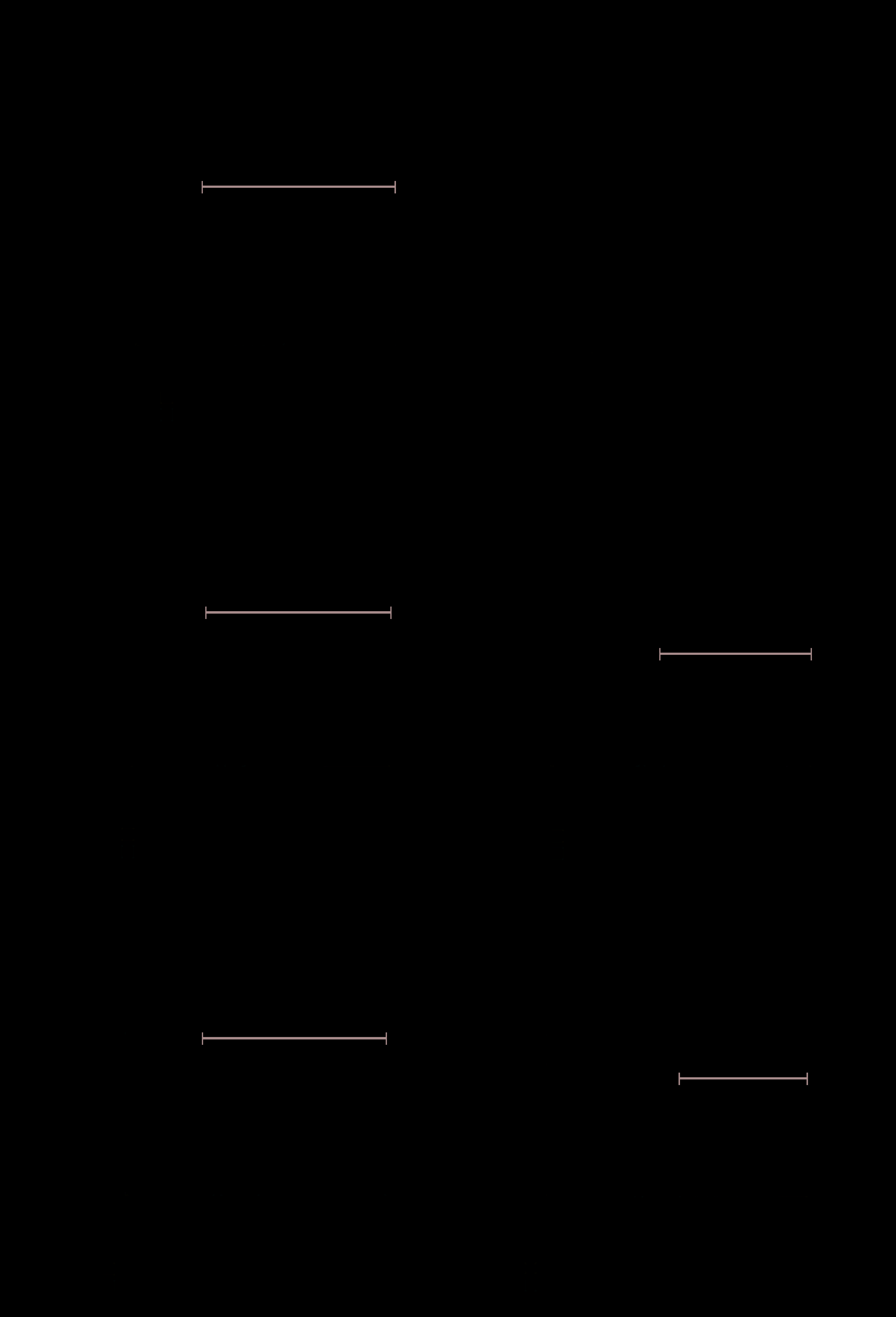

Supplement: Figure S2 — Supporting flow cytometry data for gamma delta T cell culture derived from Donor 1 isolation#11. d = culture day; GD TCR = gamma delta T cell antigen receptor; AB TCR = alpha beta T cell antigen receptor. The upper panels show purity of gamma delta T cells isolated via positive selection (MACS magnetic sorting, Miltenyi). On the upper left, gamma delta T cells are bound to beads that are FITC labeled. This fraction was also labeled with anti-AB TCR antibody (top right panel). Gating and percent positive GD TCR and AB TCR are indicated. The middle and lower panels show staining of cells harvested on culture days 12 and 18, respectively. Live cells were gated in forward and side scatter (not shown). Negative controls in red are the unstained peripheral blood mononuclear cell fraction before sorting (preMACS, top panels) or unstained cells (middle and bottom left). (TIFF) [file pone.0016700.s004.tiff]

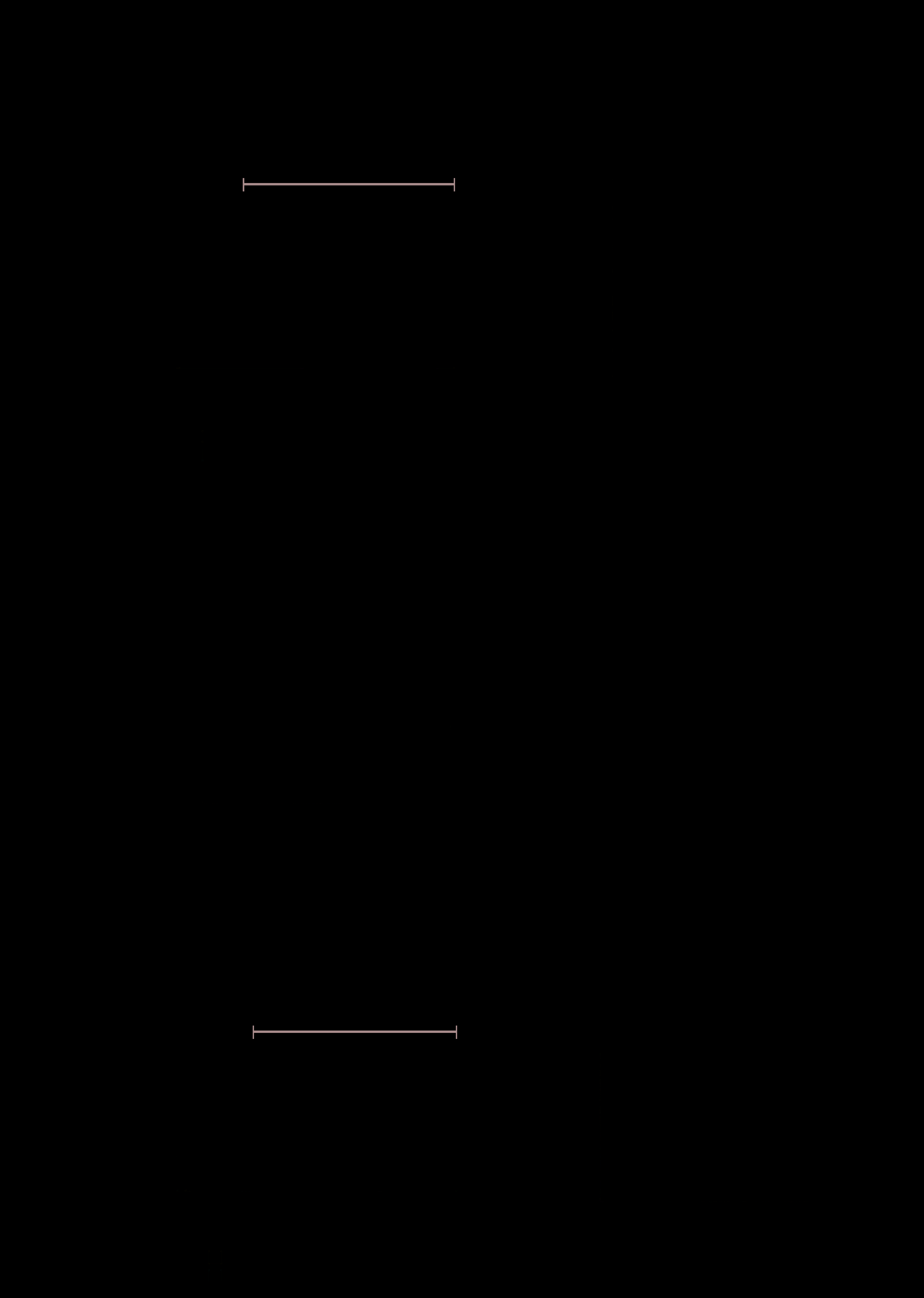

Supplement: Figure S5 — Supporting flow cytometry data for gamma delta T cell culture derived from Donor 1 isolation#34. d = culture day; GD TCR = gamma delta T cell antigen receptor; AB TCR = alpha beta T cell antigen receptor; Vdelta1 = Vdelta1 GD TCR; Vdelta2 = Vdelta2 GD TCR. The upper panels show purity of gamma delta T cells isolated via positive selection (MACS magnetic sorting, Miltenyi). On the upper left, gamma delta T cells are bound to beads that are FITC labeled. This fraction was also labeled with anti-AB TCR antibody (top right panel). Gating and percentage of cells positive for the indicated receptors are shown. The middle and lower panels show staining of cells harvested on culture days 9 and 16, respectively. Live cells were gated in forward and side scatter (not shown). Negative controls in red are the unstained peripheral blood mononuclear cell fraction before sorting (preMACS, top left panel) or unstained cells (bottom left panel). (TIFF) [file pone.0016700.s007.tiff]

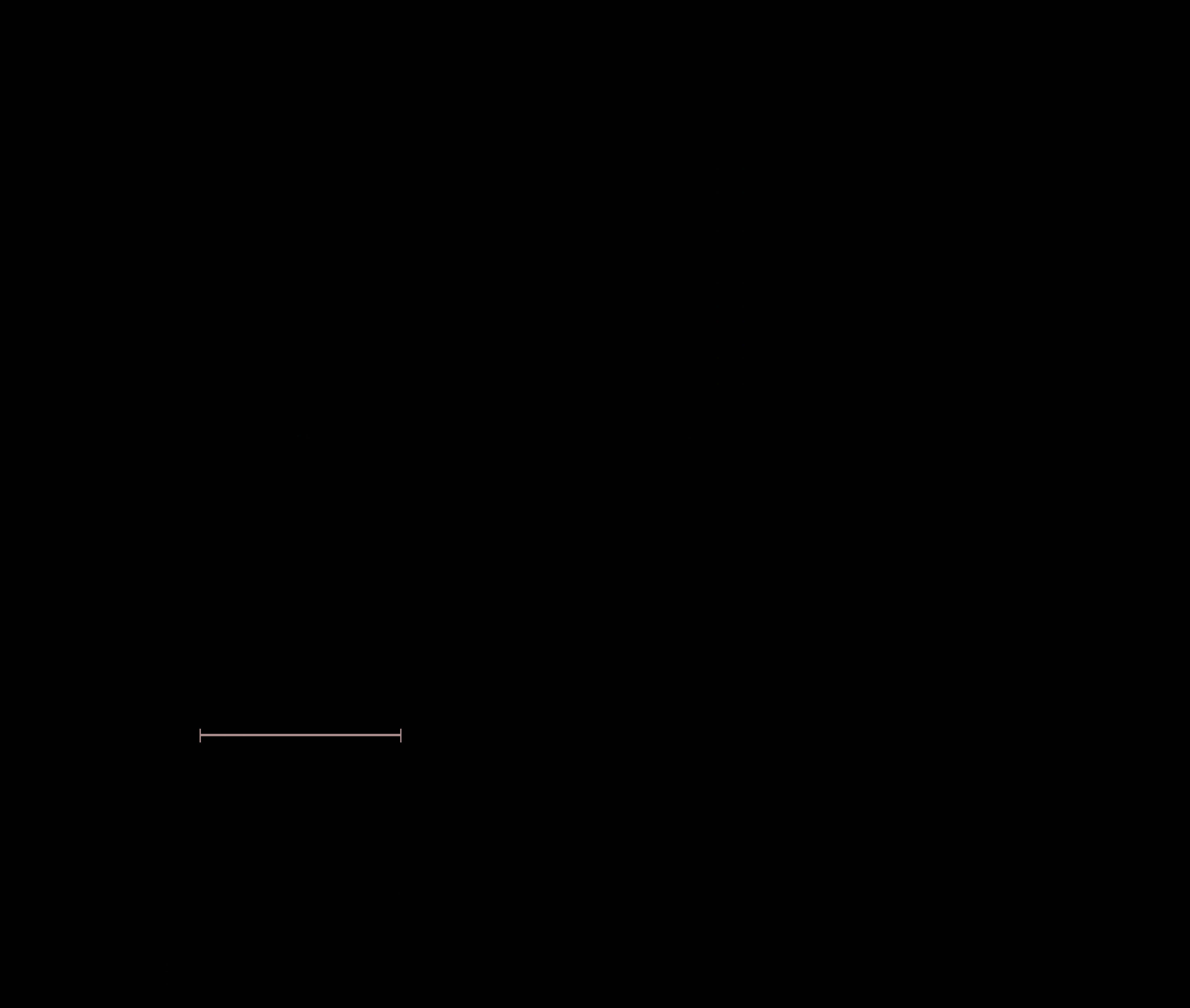

Supplement: Figure S6 — Titration of anti-CD27 APC antibody. Aliquots of the MACS negative fraction (9 x 10^5^ cells/aliquot) were stained with the indicated dilutions of anti-CD27 APC antibody in 20 µl for 20 min, washed and subject to flow cytometry. The negative control (red line) is unstained MACS negative cells. The upper panel shows histogram overlays of all dilutions tested. Mean fluorescence intensity (mfi) values are indicated. The lower panel shows overlays of the unstained control (red) and the 1:100 dilution used in Figure 1b (blue). (TIFF) [file pone.0016700.s008.tiff]

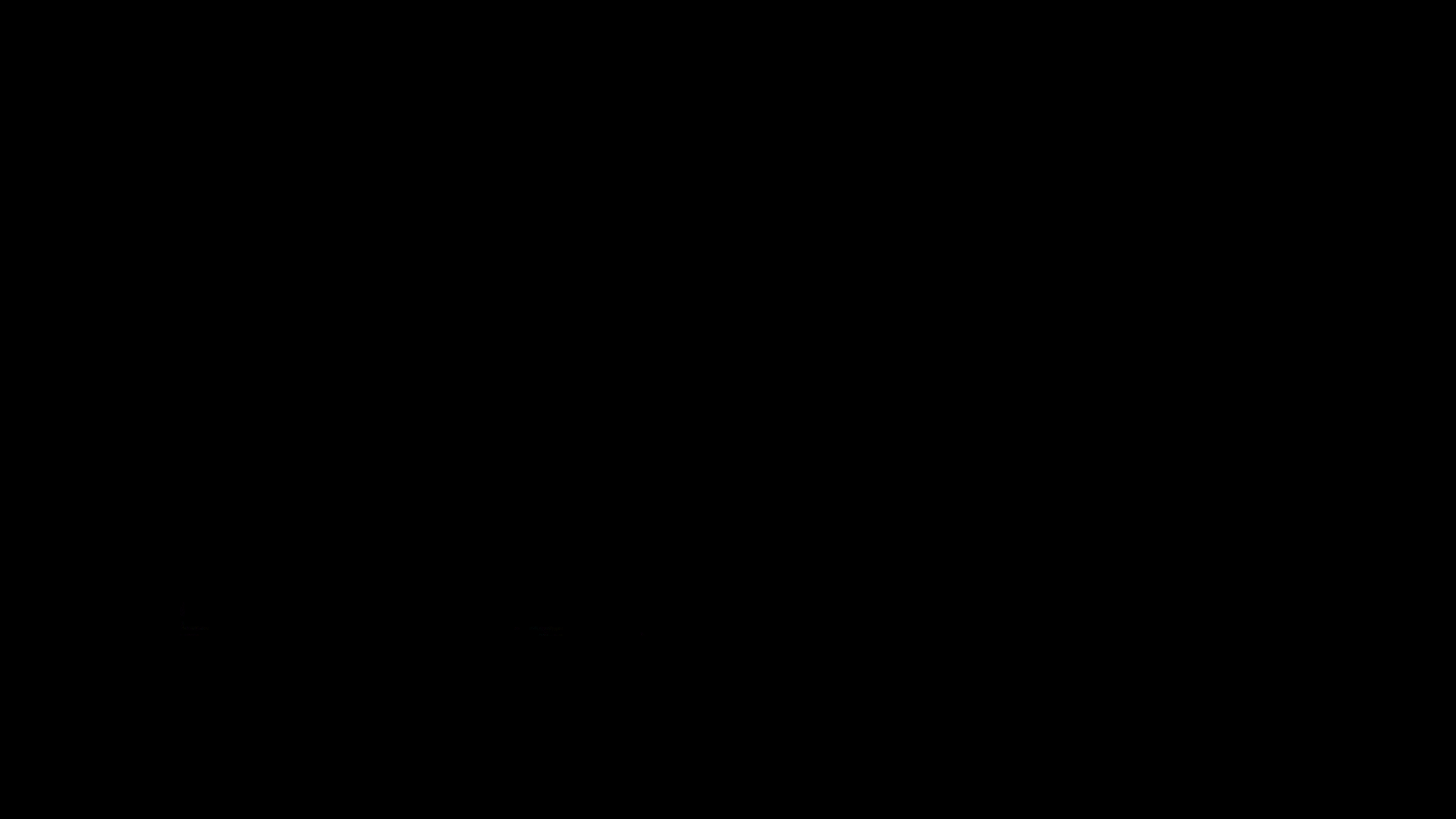

Supplement: Figure S7 — Transduced and parental leukemia cell lines elicit similar CD107 mobilization in gamma delta T cells. CD107 experiments were performed using Donor 3 gamma delta T cells and target lines EM-2 and EM-2eGFPluc. All samples had been stained using anti-human CD107aAlexa647. Live GDTc were gated; shown is a representative example, n = 3 different donors. (TIFF) [file pone.0016700.s009.tiff]

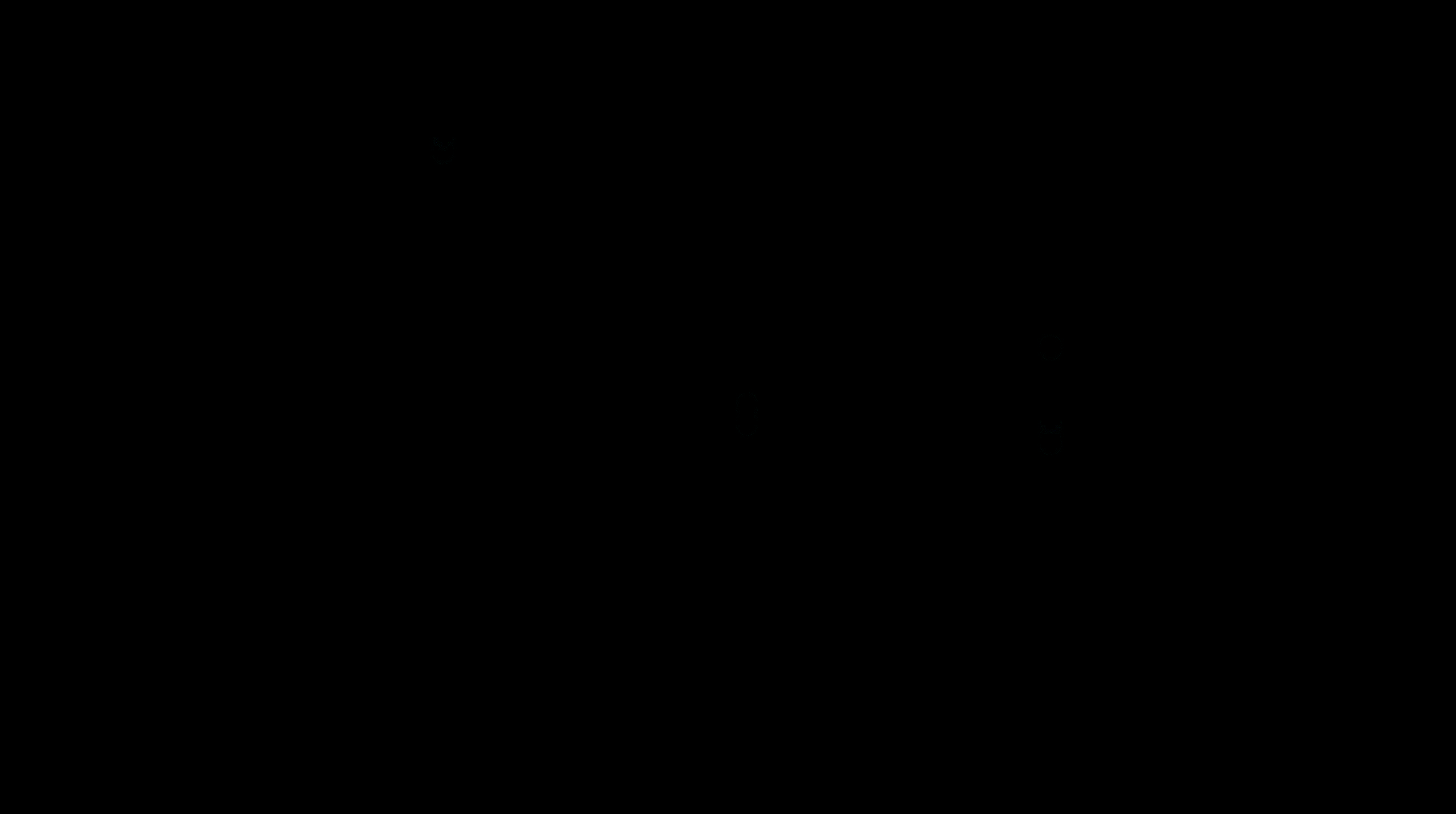

Supplement: Figure S8 — Transduced RAJI cells do not activate gamma delta T cells. CD107 experiments were performed using Donor 2 gamma delta T cells and target lines EM-2, RAJI and RAJIeGFPluc. All samples had been stained using anti-human CD107aAlexa647 and anti-Vdelta2PE antibodies. Cells were gated on live Vdelta2+ GDTc; mean fluorescence intensity values are shown in red and %CD107 in black. (TIFF) [file pone.0016700.s010.tiff]

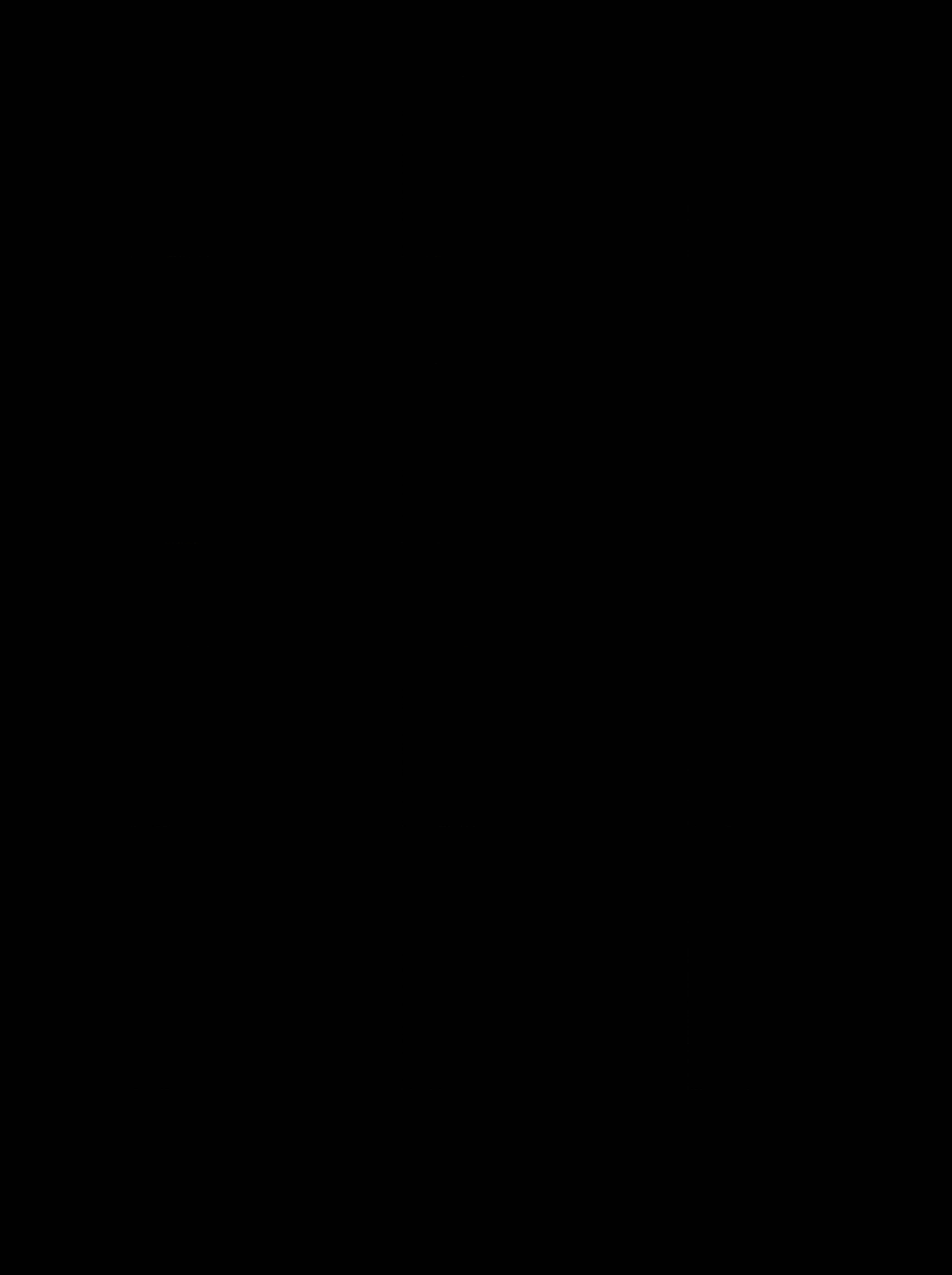

Supplement: Figure S9 — Additional bone marrow flow cytometry data from therapy experiment shown in Fig. 5c . Bone marrow from treated (T, n = 7), untreated (U, n = 1) and PBS control (n = 2) mice was extracted and stained with anti-CD3APC and anti-CD45PE antibodies and then subject to flow cytometric analysis. The live cell gate was based on forward and side scatter properties; the GFP-negative population of live cells is shown. The percentage of CD3/CD45 positive cells is indicated. * mice that received two gamma delta T cell doses. (TIFF) [file pone.0016700.s011.tiff]

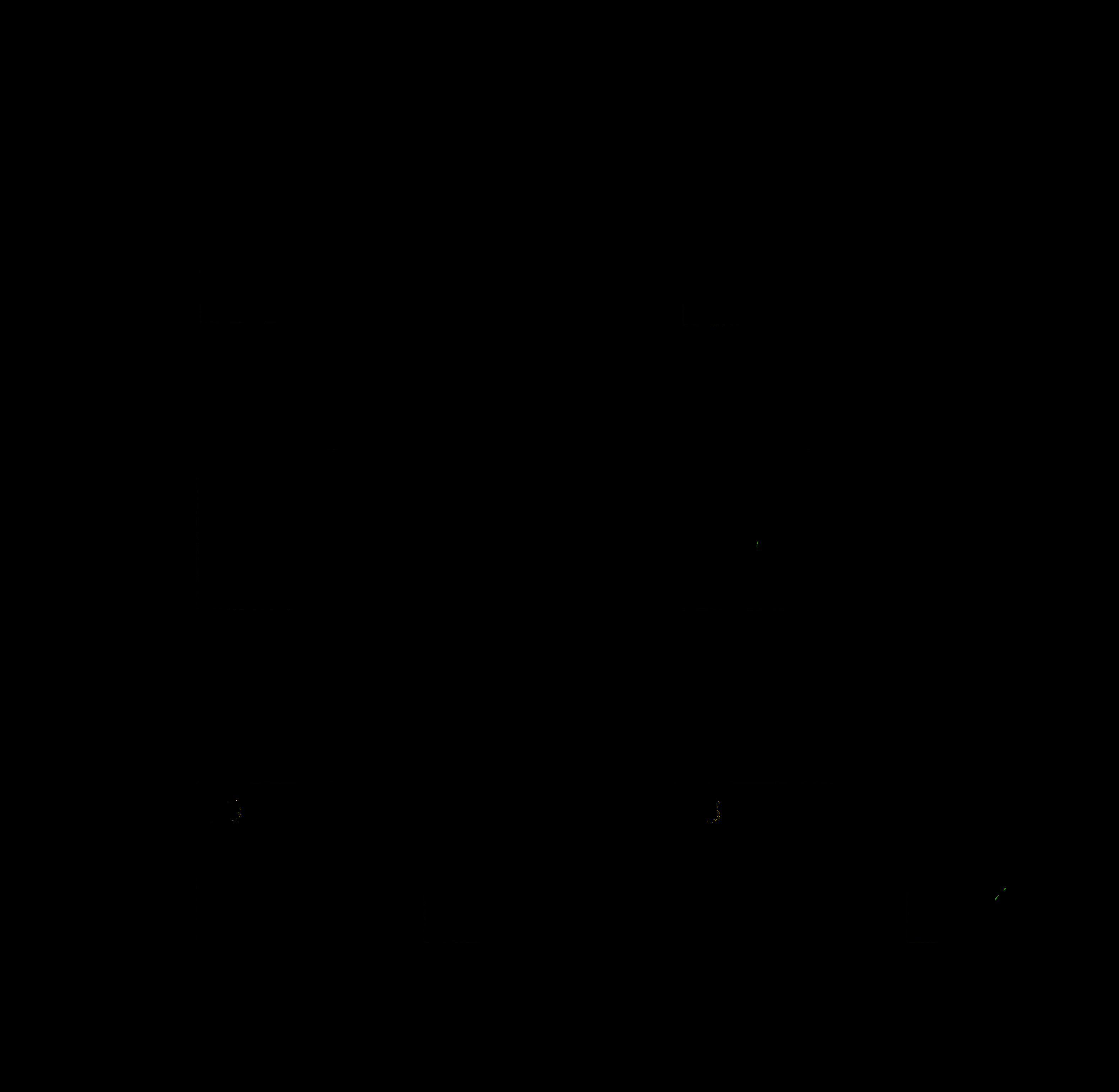

Supplement: Figure S10 — Gamma delta T cells are found in spleen, blood and bone marrow. Flow cytometric analysis showing gamma delta T cell engraftment in the tissues of one therapy mouse (#202) at endpoint in the experiment shown in Figure 6. One untreated mouse (#199) is shown as a negative control. Gates and percentages are indicated. (TIFF) [file pone.0016700.s012.tiff]
